# A multimodal feature fusion model integrating voice acoustic features and early key risk factors for early screening of postpartum depression: a prospective short-term longitudinal observational study

**DOI:** 10.3389/fpubh.2026.1906621

**Published:** 2026-08-25

**Authors:** Xuefei Han, Chongyu Yue, Xinwei Zhang, Xiaofei Ji, Shusen Lin, Meiyu Chen, Xiaojing Wang, Yanxia Zhang, Wanyu Xu, Yinhua Liu, Huawei Li

**Affiliations:** 1 School of Nursing, Qingdao University, Qingdao, Shandong, China; 2 Affiliated Hospital of Qingdao University, Qingdao, Shandong, China; 3 School of Computer Science, Qingdao University, Qingdao, Shandong, China; 4 Institute For Future, Qingdao University, Qingdao, Shandong, China

**Keywords:** early screening, machine learning, postpartum depression, SHapley Additive exPlanations, speech acoustics

## Abstract

**Background:**

Postpartum depression (PPD) is a prevalent perinatal mental health disorder, yet early identification remains challenging due to the reliance on subjective self-report screening. Voice acoustic features offer a promising objective alternative, but their utility for early PPD screening in perinatal populations is underexplored.

**Methods:**

A prospective short-term longitudinal study was conducted among 64 postpartum women (32 screening-positive, 32 screening-negative). Speech recordings and socio-ecological risk factors were collected on the day before hospital discharge, and PPD screening status was determined at 42 days postpartum using the Edinburgh Postnatal Depression Scale (EPDS ≥ 10). Sixty-five acoustic features, including mel-frequency cepstral coefficients (MFCCs), zero-crossing rate, fundamental frequency, and energy parameters, were extracted and combined with seven early key risk factors. Seven machine learning classifiers were evaluated under 10-fold GroupKFold cross-validation, and model performance was assessed using AUC, sensitivity, specificity, and related metrics. The best-performing model was interpreted using SHapley Additive exPlanations (SHAP).

**Results:**

Voice-only models substantially outperformed risk-only models across all classifiers. The fusion of voice and risk factors achieved the highest AUC of 0.871 (95% CI, 0.818–0.920) for the random forest model; however, this improvement over the best voice-only model was not statistically significant (*p* = 0.147). SHAP analysis revealed that acoustic features dominated model predictions, with MFCC and energy-related features ranking highest. Despite suboptimal probability calibration, decision curve analysis demonstrated positive net benefit across a clinically relevant range of threshold probabilities.

**Conclusion:**

Voice acoustic features collected before hospital discharge show potential as objective markers for early PPD screening, although further validation in larger, independent cohorts is required before clinical application.

## 1 Introduction

Postpartum depression (PPD) is a common perinatal mental health disorder characterized by persistent sadness, anhedonia, irritability, and hopelessness, with suicidal ideation in severe cases (1). The peak symptomatic period typically occurs 4–6 weeks postpartum (2). Globally, over 10% of pregnant and postpartum women experience PPD, with substantially higher prevalence in low- and middle-income countries (3). In China, the pooled prevalence of PPD is estimated at approximately 21.4% (4). Beyond its detrimental impact on maternal health, PPD is associated with adverse developmental, behavioral, and psychological outcomes in offspring (5), as well as increased healthcare utilization and societal burden. Without timely intervention, PPD may progress to severe major depressive disorder (6). Consequently, early identification of at-risk postpartum women and the provision of personalized psychological support are critical public health priorities.

At present, PPD screening is not routinely integrated into standard postpartum care in many settings. Diagnosis and severity assessment rely predominantly on standardized self-report scales such as the Edinburgh Postnatal Depression Scale (EPDS). Although these instruments are widely used, their diagnostic accuracy is contingent upon maternal subjective reporting and may be compromised by recall bias, symptom minimization due to stigma, or the lack of overt clinical manifestations in early stages (7, 8). Moreover, the increasing demand for perinatal mental health services, juxtaposed with constrained nursing workforce resources, poses significant challenges for comprehensive assessment. Thus, there is an urgent need to develop objective, scalable screening tools that complement and extend the capabilities of existing standardized evaluations.

Voice characteristics convey rich emotional and physiological information. Acoustic features such as fundamental frequency, spectral energy distribution, and voice quality parameters are influenced by the speaker’s affective state and are partly expressed subconsciously (9). These signals can be captured non-invasively using widely available smart devices, and recent advances in open-source audio analysis toolkits have enabled efficient extraction of high-dimensional acoustic features from short speech recordings (10). A growing body of evidence has shown that vocal acoustic features can distinguish individuals with depression from healthy controls, with pooled performance falling in the moderate-to-high range (11, 12). Mel-frequency cepstral coefficients (MFCCs) in particular have demonstrated robust discriminative capacity in speech-based emotion recognition and depression screening tasks (13, 14). Most of this work, however, has been conducted on clinical samples of major depressive disorder or university students, and little is known about the utility of voice-based screening in perinatal populations. Moreover, most existing studies derive from Western populations, and their findings may not be directly generalizable to the Chinese postpartum context due to cultural and demographic variations.

In parallel, socio-ecological frameworks have been applied to identify early key risk factors for PPD that extend beyond individual-level characteristics to encompass interpersonal, community, and societal influences. Prior work by our research team identified seven independent early key risk factors—including sleep quality, relationship with parents-in-law, social support, and spousal caregiving—that are measurable during the immediate postpartum hospitalization (15). These risk factors, while informative, are subject to the same limitations as other self-report measures. An important question is whether voice acoustic features, which can be captured objectively and passively, can complement or even substitute for questionnaire-based risk factor assessment in early PPD screening.

Machine learning (ML) methods have demonstrated superior performance in emotion classification and disease detection tasks compared to traditional statistical approaches (16). However, clinical adoption of ML models has been hindered by concerns regarding their “black-box” nature, which limits transparency and clinical actionability. To address this, post-hoc explanation methods such as SHapley Additive exPlanations (SHAP) have been developed to quantify the contribution of each feature to individual model predictions (17). The application of SHAP to voice-based PPD screening models may therefore enhance both interpretability and clinical trust.

Against this backdrop, the present study aimed to: (1) evaluate and compare the performance of seven machine learning classifiers for early PPD screening using voice acoustic features and early key socio-ecological risk factors collected before hospital discharge; (2) assess whether combining voice features with risk factors in a fusion model improves predictive performance over either modality alone; and (3) identify, through SHAP analysis, the most influential features driving model predictions.

## 2 Methods

This study was conducted and reported in accordance with the Transparent Reporting of a multivariable prediction model for Individual Prognosis or Diagnosis (TRIPOD) statement (18). The research protocol was approved by the Ethics Committee of Qingdao Medical College, Qingdao University (Approval No. QDU-HEC-2021114), and written informed consent was obtained from all participants prior to enrolment.

### 2.1 Study design and participants

A prospective short-term longitudinal observational study design was employed. Participants were recruited via convenience sampling from the obstetrics inpatient department of a tertiary Grade A hospital in Qingdao, China, between September and December 2025. Inclusion criteria were: (1) postpartum women following vaginal delivery or Cesarean section with stable clinical condition; (2) absence of severe obstetric complications (e.g., postpartum hemorrhage, amniotic fluid embolism, uterine rupture); (3) no history of psychiatric illness or intellectual disability; and (4) ability to provide informed consent and cooperate with study procedures. Exclusion criteria were: (1) concomitant severe organ failure (cardiac, hepatic, or renal), malignancy, or autoimmune disease; (2) visual impairment precluding completion of the picture description task; (3) stillbirth, neonatal death, or fetal congenital anomalies; and (4) experience of major familial trauma or adversity between pregnancy and the postpartum period. All predictor variables—speech recordings and early key risk factor screening—were collected on the day before hospital discharge. The outcome, PPD screening status, was determined at 42 days postpartum using the Chinese version of the Edinburgh Postnatal Depression Scale (EPDS). A cut-off score of ≥10 was employed to define screening-positive cases (19). This temporal separation between predictor collection and outcome ascertainment constitutes the “early prediction” framework of the present study.

Sample size estimation was guided by the expected number of PPD cases required for model development, given the absence of a publicly available multimodal dataset for postpartum depression. Based on the reported depression case counts in existing multimodal depression datasets—specifically, 23 cases in the MODMA dataset (20) and 30 cases in the EATD-Corpus (21)—a minimum of 30 PPD-positive cases was targeted. With an estimated PPD prevalence of 21.4% among Chinese postpartum women (4), a minimum of 141 participants (30/0.214) was required. Accounting for a potential 20% attrition rate due to dropout or data quality exclusion—a rate consistent with general recommendations for longitudinal studies involving speech recordings, in which data loss may result from recording artifacts, incomplete tasks, or withdrawal (22, 23)—the recruitment target was set at 176 participants. A total of 192 postpartum women were ultimately enrolled, yielding 38 PPD-positive cases, which satisfied the prespecified minimum requirement.

Following quality control and exclusion of six screening-positive cases with poor-quality recordings, 32 screening-positive and 32 screening-negative participants (randomly selected from the eligible negative pool to ensure class balance) were retained for model development, yielding a final analytic sample of 64 participants.

### 2.2 Instruments and measures

#### 2.2.1 Early key risk factor screening form for PPD

The early key risk factor screening form for postpartum depression was derived from prior work by our research team (15). Briefly, that study applied a social-ecological framework to collect 53 candidate risk factors from 569 postpartum women. Univariate and multivariate logistic regression analyses identified seven independent early key risk factors for PPD: maternal appraisal of the postpartum recuperation environment, relationship with parents-in-law, level of social support, postpartum sleep quality, work-related stress during maternity leave, adequacy of spousal caregiving postpartum, and distress related to gestational weight gain. These seven items constituted the early key risk factor component of the multimodal feature set.

#### 2.2.2 Chinese affective picture system (CAPS)

The CAPS, developed by the Institute of Psychology, Chinese Academy of Sciences (24), is a standardized set of emotional picture stimuli widely employed in psychological research and clinical emotion assessment. Evidence suggests that individuals with depression exhibit cognitive biases characterized by negative interpretations of neutral or even positive stimuli (25, 26). Moreover, the type of speech task influences emotion recognition performance, with picture description tasks outperforming reading tasks (27). Accordingly, three representative images were selected from CAPS following expert consultation: image 756 (positive valence), image 715 (neutral valence), and image 229 (negative valence). During the task, images were presented sequentially, and participants were instructed to describe the content orally, thereby eliciting emotionally expressive behavioral responses.

#### 2.2.3 Chinese version of the Edinburgh postnatal depression scale (EPDS)

The EPDS, originally developed by Cox et al. (28), is a 10-item self-report instrument designed to assess depressive symptoms over the preceding week. Items are rated on a 4-point Likert scale (0–3), yielding a total score ranging from 0 to 30. It is the most widely used screening tool for postpartum depression and is typically administered at 6 weeks postpartum (28). The Chinese version, validated by Lee et al. (29), has demonstrated sound psychometric properties in Chinese populations. In the present study, the EPDS was administered at 42 days postpartum to determine PPD screening status. A cut-off score of ≥10 was employed to define screening-positive cases. This threshold is the most commonly used cut-off in perinatal care and has demonstrated a sensitivity of 0.85 and a specificity of 0.84 for detecting major depression among pregnant and postpartum women in a large individual participant data meta-analysis (19). Cronbach’s alpha in this sample was 0.880, indicating good internal consistency.

### 2.3 Data collection and quality control

Data acquisition was performed between 9:30 a.m. and 4:00 p.m. on the day before maternal discharge. Recordings took place in a quiet private room within the obstetrics ward, with ambient noise monitored and maintained below 40 dB throughout each session. Participants spoke facing a tablet device (Xiaomi Pad 7 Pro; Xiaomi Corporation, Beijing, China) placed at a fixed distance of approximately 20 cm on a stand. Audio was captured using the tablet’s built-in microphone at a sampling rate of 44.1 kHz with 16-bit resolution and saved in uncompressed WAV format. A trained researcher guided each participant through the following sequence: (1) completion of the early key risk factor screening form; (2) a picture description task with simultaneous audio recording. All tasks were administered via a custom-designed application developed by our research team and deployed on the tablet device, enabling synchronized collection of screening form responses and speech audio. Outcome assessment using the EPDS was conducted independently at 42 days postpartum.

A total of 192 postpartum women were initially enrolled with complete speech and risk factor data. Two trained researchers independently reviewed all recordings to exclude samples with excessive background noise, persistent artifacts, or incomplete utterances. Non-speech segments—including prolonged silences before, after, or within utterances—were manually identified and removed to ensure that only voiced portions were retained for analysis. After quality control, six screening-positive cases were excluded. To construct a balanced dataset for model training, 32 participants were randomly selected from the eligible screening-negative pool using a fixed random seed, yielding a final analytic sample of 64 participants (32 screening-positive, 32 screening-negative). The resulting clean speech segments were then used for subsequent acoustic feature extraction.

### 2.4 Speech feature extraction

To capture vocal affective characteristics associated with PPD, a comprehensive set of acoustic features was extracted using the open-source Librosa audio analysis toolkit (10). The feature set comprised: (1) Mel-frequency cepstral coefficients (MFCCs 1–13), which effectively characterize vocal tract filter properties and have demonstrated robust discriminative capacity in speech emotion recognition tasks (13, 30); (2) zero-crossing rate (ZCR), which reflects the distribution of voiced and unvoiced segments as well as noise levels, thereby facilitating emotion detection from speech (31); (3) fundamental frequency (F0), which represents the periodicity of vocal fold vibration, with variations indicative of the speaker’s emotional state (e.g., low mood, tension) (32); (4) root mean square energy (RMS), which quantifies speech loudness and serves as an important index of emotional arousal (33); and (5) harmonics-to-noise ratio (HNR), a measure of voice quality that quantifies the relative level of harmonic energy to noise, often associated with vocal tension, breathiness, and hoarseness (34). A frame-based analysis was employed (frame length: 20 ms; frame shift: 10 ms) (35). For MFCCs, ZCR, F0, and RMS, the mean, standard deviation, maximum, and minimum values were calculated across the entire utterance. Unlike frame-level descriptors, HNR was computed as a single global value for the entire utterance, reflecting the overall ratio of harmonic to noise energy. Therefore, only the mean HNR was retained, yielding a final total of 65 acoustic features. A detailed summary of all extracted features is provided in Table 1.

**Table 1:** Summary of extracted acoustic features.

Feature group	Feature (statistic)	Count	Explanation
Spectral		52	
	MFCC 1–13 (Mean)	13	Mean of the first 13 mel-frequency cepstral coefficients, characterizing the overall shape of the vocal tract spectrum.
	MFCC 1–13 (Std)	13	Standard deviation of the 13 MFCCs, reflecting the temporal variability of the vocal tract configuration.
	MFCC 1–13 (Max)	13	Maximum value of the 13 MFCCs, capturing extreme spectral energy distributions.
	MFCC 1–13 (Min)	13	Minimum value of the 13 MFCCs, capturing the lower bounds of spectral energy.
Voice quality		1	
	HNR (Mean)	1	Mean Harmonics-to-Noise Ratio, quantifying the relative level of harmonic energy to noise. Associated with vocal tension, breathiness, and hoarseness.
Prosodic/Energy			
	ZCR (Mean, Std, Max, Min)	4	Statistics of the Zero-Crossing Rate, reflecting the distribution of voiced and unvoiced segments and high-frequency noise.
	F0 (Mean, Std, Max, Min)	4	Statistics of the Fundamental Frequency (pitch), representing the periodicity of vocal fold vibration. Variations are indicative of emotional state.
	RMS (Mean, Std, Max, Min)	4	Statistics of the Root Mean Square energy, quantifying speech loudness and serving as an index of emotional arousal.
Total		65	

MFCC, Mel-Frequency Cepstral Coefficients; HNR, Harmonics-to-Noise Ratio; ZCR, Zero-Crossing Rate; F0, Fundamental Frequency; RMS, Root Mean Square energy; Std, standard deviation; Max, maximum; Min, minimum. The first 13 MFCCs (MFCC 1 to MFCC 13, corresponding to coefficients 0 to 12 in the Librosa implementation) were extracted.

### 2.5 Feature integration and normalization

After extraction, the 65 acoustic features were directly concatenated with the 7 early key risk factors to form a 72-dimensional feature vector through simple feature-level fusion. Since each participant contributed three speech samples (positive, neutral, and negative picture descriptions), the risk factor values, which are participant-level characteristics, were replicated across the three corresponding speech samples. All features were standardized using z-score normalization to ensure a mean of zero and unit variance, thereby mitigating scaling disparities among features and reducing computational burden during model training (36).

### 2.6 Statistical analysis

Clinical and demographic characteristics were compared between the PPD-positive and PPD-negative groups within the final analytic sample of 64 participants. Categorical variables are presented as frequencies and percentages, with between-group comparisons performed using the chi-square test or Fisher’s exact test as appropriate. Symmetrically distributed continuous variables are expressed as mean ± standard deviation (SD) and compared using the independent samples t-test. Skewed continuous variables are presented as median and interquartile range (IQR) and compared using the Mann–Whitney U test. A two-tailed *p*-value < 0.05 was considered statistically significant. All statistical analyses were performed using SPSS version 27.0.

### 2.7 Machine learning model development and evaluation

Machine learning modeling was implemented in Python 3.12. Consistent with recent empirical research (35), seven commonly used supervised classifiers were evaluated: logistic regression (LR), support vector machine (SVM) with linear kernel, random forest (RF), K-nearest neighbors (KNN), Gaussian naive Bayes (NB), gradient boosting (GB), and XGBoost. No formal feature selection or dimensionality reduction was applied prior to model training. All 72 features were included in the fusion configuration to allow the classifiers to learn from the full set of available information. Models were trained using their default hyperparameter settings, with the following adjustments to control overfitting: the random forest and gradient boosting classifiers used a maximum tree depth of 5, and XGBoost used a maximum depth of 3 with a learning rate of 0.1. No further hyperparameter optimization was performed, as the use of default or fixed hyperparameters reduces the risk of over-optimism associated with extensive grid or random search in small samples. Three distinct feature configurations were constructed to assess the individual and combined contributions of voice acoustics and early key risk factors: (1) voice only (65 acoustic features); (2) risk factors only (7 socio-ecological items); and (3) fusion (all 72 features).

Model performance was assessed using the area under the receiver operating characteristic curve (AUC) as the primary metric. Secondary metrics included accuracy, sensitivity (recall), specificity, and F1-score. For all metrics, 95% confidence intervals (CIs) were estimated via bootstrap resampling (1,000 iterations) on the aggregated predictions across the cross-validation folds. To preserve subject independence, a 10-fold GroupKFold cross-validation strategy was employed, using participant ID as the grouping variable. This ensured that all three speech samples from a given participant were strictly confined to the same fold, preventing data leakage between the training and test sets. Within each fold, feature standardization (z-score normalization) was applied to the training data, and the learned parameters were then used to transform the test data, ensuring that no information from the test set influenced the training process. Given that each participant contributed three speech samples (positive, neutral, and negative picture descriptions), which are inherently correlated within the same individual, a 10-fold GroupKFold cross-validation strategy was employed, using participant ID as the grouping variable. This approach ensured that all samples from a given participant were strictly confined to the same fold, thereby preventing data leakage between the training and test sets and preserving subject independence. Within each training fold, features were standardized to zero mean and unit variance. All models were trained using fixed, default hyperparameters without further tuning, to avoid over-optimism and ensure a fair comparison across classifiers.

The best-performing model was selected for subsequent calibration and interpretability analyses. Predicted probabilities were calibrated using Platt scaling (sigmoid method). Calibration quality was evaluated using the Hosmer–Lemeshow goodness-of-fit test and calibration plots. The clinical net benefit of the calibrated model across a range of threshold probabilities was assessed using decision curve analysis (DCA). To formally compare the AUC values across the three feature configurations, the non-parametric Friedman test was used, followed by the post-hoc Nemenyi test for pairwise comparisons. A two-tailed *p*-value < 0.05 was considered statistically significant.

### 2.8 Model interpretability

To address the “black-box” nature of machine learning models and enhance clinical actionability, SHapley Additive exPlanations (SHAP) were employed to interpret the best-performing model (17). SHAP values, which quantify the marginal contribution of each feature to individual predictions, were computed for all participants. Global feature importance was derived by averaging absolute SHAP values across the dataset. The top 20 most influential features were visualized using an integrated polar coordinate plot and rose diagram, which simultaneously display the global importance ranking (via radial bars) and the distribution of individual-level SHAP values (via beeswarm scatter points), thereby illustrating both the magnitude and direction of each feature’s effect on model predictions.

**Figure 1:**
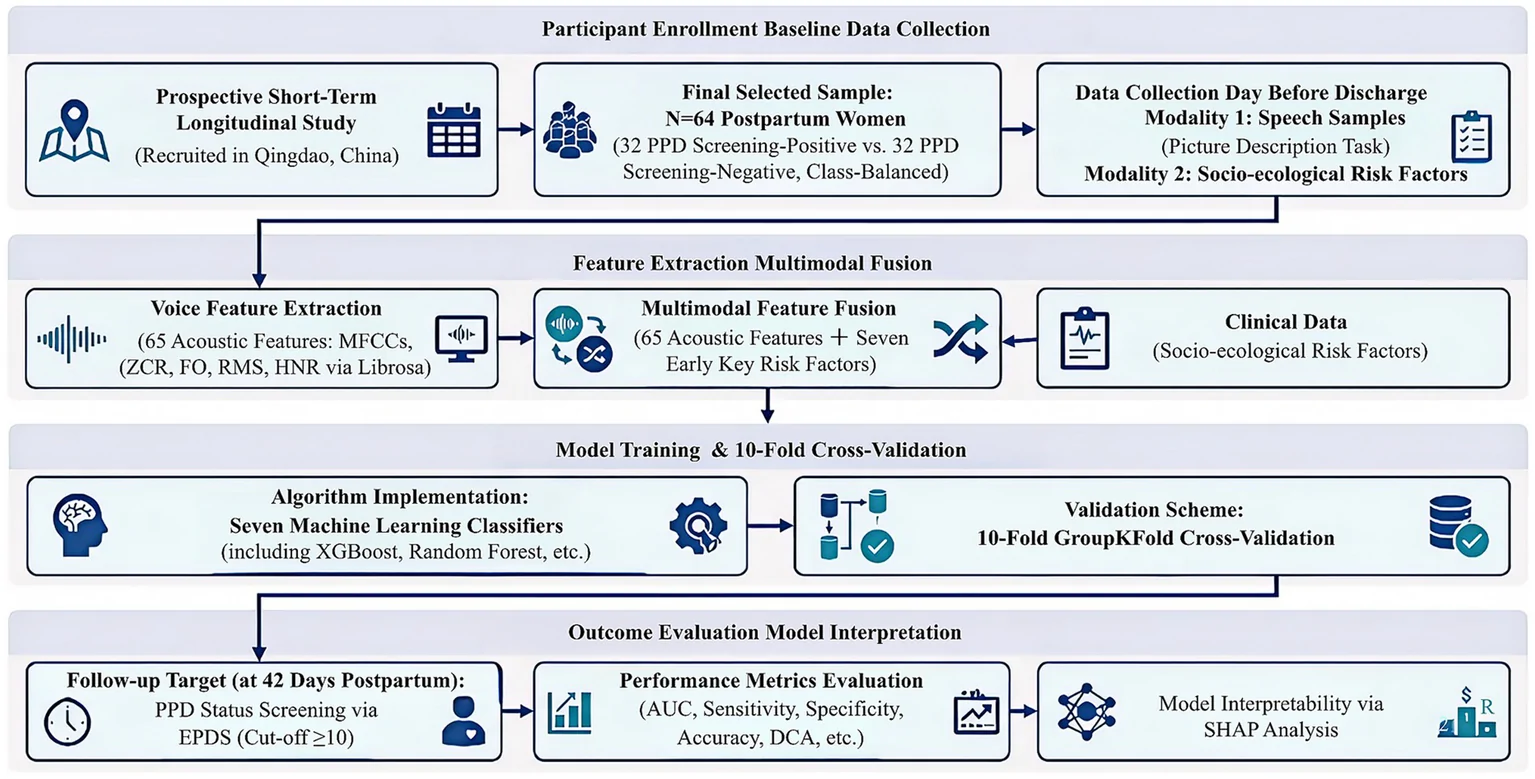
Overview of the study workflow. (Step 1) On the day before hospital discharge, speech recordings were collected via a picture description task and early key socio-ecological risk factors were assessed. The final analytic sample comprised 64 participants (32 PPD screening-positive, 32 screening-negative). (Step 2) A total of 65 acoustic features—including mel-frequency cepstral coefficients (MFCCs), zero-crossing rate (ZCR), fundamental frequency (F0), root mean square energy (RMS), and harmonics-to-noise ratio (HNR)—were extracted using Librosa and combined with seven socio-ecological risk factors. (Step 3) Seven machine learning classifiers were trained and evaluated under 10-fold GroupKFold cross-validation. (Step 4) PPD screening status was determined at 42 days postpartum using the Edinburgh Postnatal Depression Scale (EPDS ≥ 10). Model performance was assessed, and clinical explainability was examined using SHapley Additive exPlanations (SHAP). PPD, postpartum depression; MFCCs, mel-frequency cepstral coefficients; ZCR, zero-crossing rate; F0, fundamental frequency; RMS, root mean square energy; HNR, harmonics-to-noise ratio; EPDS, Edinburgh Postnatal Depression Scale; AUC, area under the receiver operating characteristic curve; DCA, decision curve analysis; SHAP, SHapley Additive exPlanations.

The overall technical workflow of the study is presented in Figure 1.

## 3 Results

### 3.1 Participant characteristics

The final analytic sample comprised 64 postpartum women: 32 who screened positive for PPD (EPDS ≥10 at 42 days postpartum) and 32 who screened negative. Demographic and clinical characteristics of the two groups are summarized in Table 2. There were no statistically significant differences between the screening-positive and screening-negative groups in age, educational level, parity, mode of delivery, infant sex, or any other baseline variable (all *p* > 0.05), confirming that the groups were well-matched on potential confounders.

**Table 2:** Baseline characteristics of participants stratified by postpartum depression screening status (positive vs. negative screening result).

Characteristic	Positive group (*n* = 32)	Negative group (*n* = 32)	Statistic	*p*-value
Age (years)	30.31 ± 3.83	31.97 ± 4.32	*t* = −1.622	0.110
Body weight (kg)	71.57 ± 8.52	73.32 ± 8.01	*t* = −0.850	0.399
Height (cm)	163.81 ± 4.89	163.97 ± 4.49	*t* = −0.133	0.894
Education level, *n* (%)			*χ^2^* < 0.001	>0.99
Junior high school or below	–	–		
Senior high school/technical secondary school	1 (3.1)	–		
College degree or above	31 (96.9)	32 (100.0)		
Primipara, *n* (%)			–^*^	0.616
Yes	19 (59.4)	16 (50.0)		
No	13 (40.6)	16 (50.0)		
Residence, *n* (%)			–^*^	0.708
Urban	27 (84.4)	29 (90.6)		
Rural	5 (15.6)	3 (9.4)		
Infant gender, *n* (%)			*χ^2^* = 1.787	0.409
Male	16 (50.0)	19 (59.4)		
Female	15 (46.9)	13 (40.6)		
Two girls	1 (3.1)	–		
Two boys	–	–		
One boy and one girl	–	–		
Infant health status, *n* (%)			–^*^	>0.99
Good	29 (90.6)	29 (90.6)		
Fair	3 (9.4)	3 (9.4)		
Marital status, *n* (%)			–^*^	>0.99
Married	32 (100.0)	31 (96.9)		
Unmarried	–	1 (3.1)		
Delivery mode, *n* (%)			–^*^	0.799
Vaginal delivery	14 (43.8)	12 (37.5)		
Cesarean section	18 (56.2)	20 (62.5)		
Gestational complications, *n* (%)			–^*^	>0.99
Yes	31 (96.9)	32 (100.0)		
No	1 (3.1)	–		
Employment status, *n* (%)			–^*^	0.492
Employed	30 (93.8)	32 (100.0)		
Unemployed	2 (6.2)	–		
Payment method, *n* (%)			*χ^2^* = 0.069	0.792
Self-paid	22 (68.8)	20 (62.5)		
Maternity insurance	10 (31.2)	12 (37.5)		

1. Continuous data: mean ± standard deviation (SD); compared by independent samples *t*-test. 2. Categorical data: *n* (%); compared by Likelihood ratio *χ*^2^ test or Fisher’s exact test (^*^Fisher’s exact test was used due to expected frequency <5). 3. *p* < 0.05 was considered statistically significant.

### 3.2 Model performance across feature configurations

**Figure 2:**
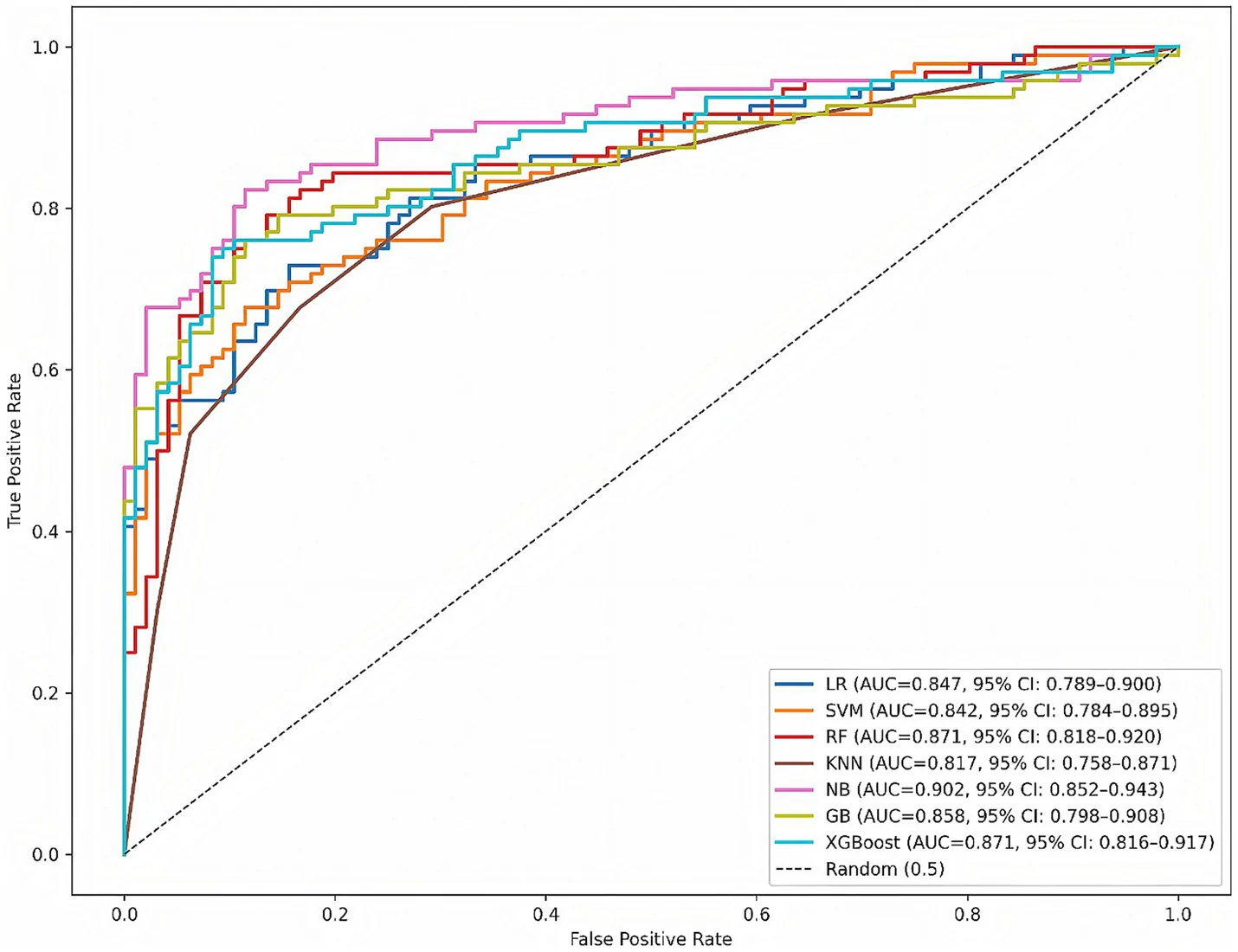
Receiver operating characteristic (ROC) curves of the seven classifiers evaluated on the fusion feature set under 10-fold GroupKFold cross-validation. The diagonal dashed line represents the performance of a random classifier (AUC = 0.50). AUC values and 95% confidence intervals (in parentheses) are shown in the legend. LR, logistic regression; SVM, support vector machine; RF, random forest; KNN, *k*-nearest neighbors; NB, Naive Bayes; GB, gradient boosting; XGBoost, extreme gradient boosting.

The discriminative performance of all seven classifiers under the three feature configurations (voice-only, risk-only, and fusion) is presented in Table 3. For visual comparison, the ROC curves of the fusion models are shown in Figure 2.

**Table 3:** Performance of seven classifiers across three feature configurations.

Model	Data	AUC (95% CI)	Accuracy (95% CI)	Sensitivity (95% CI)	Specificity (95% CI)	F1 (95% CI)
LR	Voice	0.810 (0.751–0.868)	0.741 (0.682–0.797)	0.729 (0.637–0.815)	0.752 (0.670–0.832)	0.736 (0.663–0.800)
	Risk	0.778 (0.709–0.837)	0.750 (0.688–0.807)	0.689 (0.600–0.774)	0.811 (0.732–0.887)	0.733 (0.658–0.796)
	Fusion	0.847 (0.789–0.900)	0.751 (0.693–0.812)	0.741 (0.646–0.824)	0.761 (0.677–0.842)	0.747 (0.674–0.813)
SVM	Voice	0.779 (0.712–0.842)	0.715 (0.651–0.781)	0.720 (0.626–0.802)	0.710 (0.625–0.796)	0.715 (0.641–0.782)
	Risk	0.755 (0.687–0.817)	0.640 (0.573–0.703)	0.531 (0.426–0.625)	0.749 (0.663–0.830)	0.594 (0.503–0.679)
	Fusion	0.842 (0.784–0.895)	0.745 (0.682–0.802)	0.761 (0.677–0.847)	0.730 (0.646–0.812)	0.748 (0.678–0.811)
RF	Voice	0.860 (0.800–0.911)	0.815 (0.755–0.865)	0.794 (0.714–0.870)	0.835 (0.755–0.906)	0.810 (0.740–0.865)
	Risk	0.778 (0.708–0.845)	0.750 (0.693–0.807)	0.688 (0.594–0.778)	0.811 (0.732–0.885)	0.732 (0.658–0.798)
	Fusion	**0.871 (0.818–0.920)**	**0.825 (0.766–0.880)**	**0.804 (0.721–0.875)**	**0.845 (0.767–0.918)**	**0.820 (0.756–0.878)**
KNN	Voice	0.791 (0.724–0.846)	0.736 (0.672–0.797)	0.688 (0.594–0.780)	0.783 (0.693–0.866)	0.721 (0.644–0.790)
	Risk	0.520 (0.445–0.596)	0.499 (0.427–0.573)	0.844 (0.768–0.913)	0.156 (0.090–0.230)	0.626 (0.555–0.695)
	Fusion	0.817 (0.758–0.871)	0.757 (0.698–0.813)	0.679 (0.579–0.773)	0.835 (0.762–0.906)	0.735 (0.654–0.804)
NB	Voice	0.863 (0.802–0.917)	0.762 (0.703–0.818)	0.617 (0.511–0.711)	0.907 (0.851–0.964)	0.720 (0.635–0.796)
	Risk	0.797 (0.729–0.855)	0.642 (0.573–0.714)	0.283 (0.190–0.376)	1.000 (1.000–1.000)	0.440 (0.319–0.547)
	Fusion	0.902^*^ (0.852–0.943)	0.721 (0.651–0.786)	0.441 (0.340–0.540)	1.000 (1.000–1.000)	0.610 (0.507–0.701)
GB	Voice	0.855 (0.796–0.904)	0.804 (0.745–0.854)	0.784 (0.695–0.857)	0.825 (0.750–0.892)	0.799 (0.726–0.853)
	Risk	0.825 (0.766–0.879)	0.719 (0.656–0.781)	0.626 (0.531–0.719)	0.811 (0.732–0.885)	0.689 (0.608–0.764)
	Fusion	0.858 (0.798–0.908)	0.814 (0.755–0.865)	0.793 (0.714–0.870)	0.835 (0.758–0.908)	0.809 (0.744–0.863)
XGBoost	Voice	0.864 (0.806–0.911)	0.773 (0.708–0.828)	0.762 (0.677–0.847)	0.783 (0.703–0.857)	0.769 (0.698–0.831)
	Risk	0.772 (0.702–0.839)	0.750 (0.688–0.807)	0.719 (0.627–0.806)	0.780 (0.702–0.860)	0.740 (0.662–0.805)
	Fusion	0.871 (0.816–0.917)	0.798 (0.740–0.854)	0.782 (0.702–0.863)	0.813 (0.732–0.886)	0.793 (0.730–0.852)

LR, Logistic Regression; SVM, Support Vector Machine; RF, Random Forest; KNN, K-Nearest Neighbors; NB, Gaussian Naive Bayes; GB, Gradient Boosting. Voice, voice-only features (65 acoustic features); Risk, early key risk factors (7 items); Fusion, combined features (72 features). All metrics were evaluated under 10-fold GroupKFold cross-validation. 95% CIs were estimated via bootstrap (1,000 resamples). The best AUC in each configuration is bolded. The overall best model (NB-Fusion) is marked with an asterisk (*), however, due to its low sensitivity (0.441), RF-Fusion is recommended as the most clinically balanced model (bolded row for RF-Fusion). Data are presented with 95% CI in parentheses.

#### 3.2.1 Risk-only models

When trained exclusively on the seven early key risk factors, classifiers demonstrated moderate predictive ability. The area under the ROC curve (AUC) for risk-only models ranged from 0.520 (KNN) to 0.825 (GB). The Gradient Boosting (GB) risk-only model achieved an AUC of 0.825 (95% CI, 0.766–0.879), with a sensitivity of 0.626 and specificity of 0.811. These findings are consistent with prior work indicating that socio-ecological risk factors carry meaningful, clinically relevant information for PPD screening.

#### 3.2.2 Voice-only models

Classifiers using only the 65 acoustic features exhibited improved performance over their risk-only counterparts across all seven algorithms. Voice-only AUCs ranged from 0.779 (SVM) to 0.864 (XGBoost). The Random Forest (RF) voice-only model attained an AUC of 0.860 (95% CI, 0.800–0.911), with a sensitivity of 0.794 and specificity of 0.835, while the XGBoost voice-only model reached an AUC of 0.864 (95% CI, 0.806–0.911). A sensitivity analysis using only the top 10 most important acoustic features identified by SHAP (Supplementary Table S1) showed that model performance did not deteriorate when reducing features from 65 to 10. For instance, the RF model achieved an AUC of 0.891 (95% CI, 0.840–0.932) with only 10 features, compared with 0.860 (95% CI, 0.800–0.911) with the full set, confirming the robustness of the voice-only models.

#### 3.2.3 Fusion models

The fusion of voice acoustics and risk factors yielded the highest overall AUC values. Among all models, the Naive Bayes (NB) fusion model produced the numerically highest AUC of 0.902 (95% CI, 0.852–0.943); however, its sensitivity was only 0.441, with specificity of 1.000, indicating a conservative decision boundary that heavily favored the negative class. The RF-Fusion model achieved an AUC of 0.871 (95% CI, 0.818–0.920), with a more balanced sensitivity of 0.804 and specificity of 0.845. Similarly, XGBoost-Fusion achieved an AUC of 0.871 (95% CI, 0.816–0.917). Across all classifiers, the fusion configuration consistently produced the highest AUCs, though the incremental gain over voice-only models was modest in magnitude. A Friedman test confirmed a significant overall difference among the three feature configurations (*χ^2^* = 14.000, df = 2, *p* < 0.001). Post-hoc Nemenyi tests revealed that the fusion configuration significantly outperformed the risk-only configuration (*p* < 0.001), while the differences between voice-only and risk-only (*p* = 0.147), and between fusion and voice-only (*p* = 0.147), were not statistically significant.

### 3.3 Feature importance and interpretability

**Figure 3:**
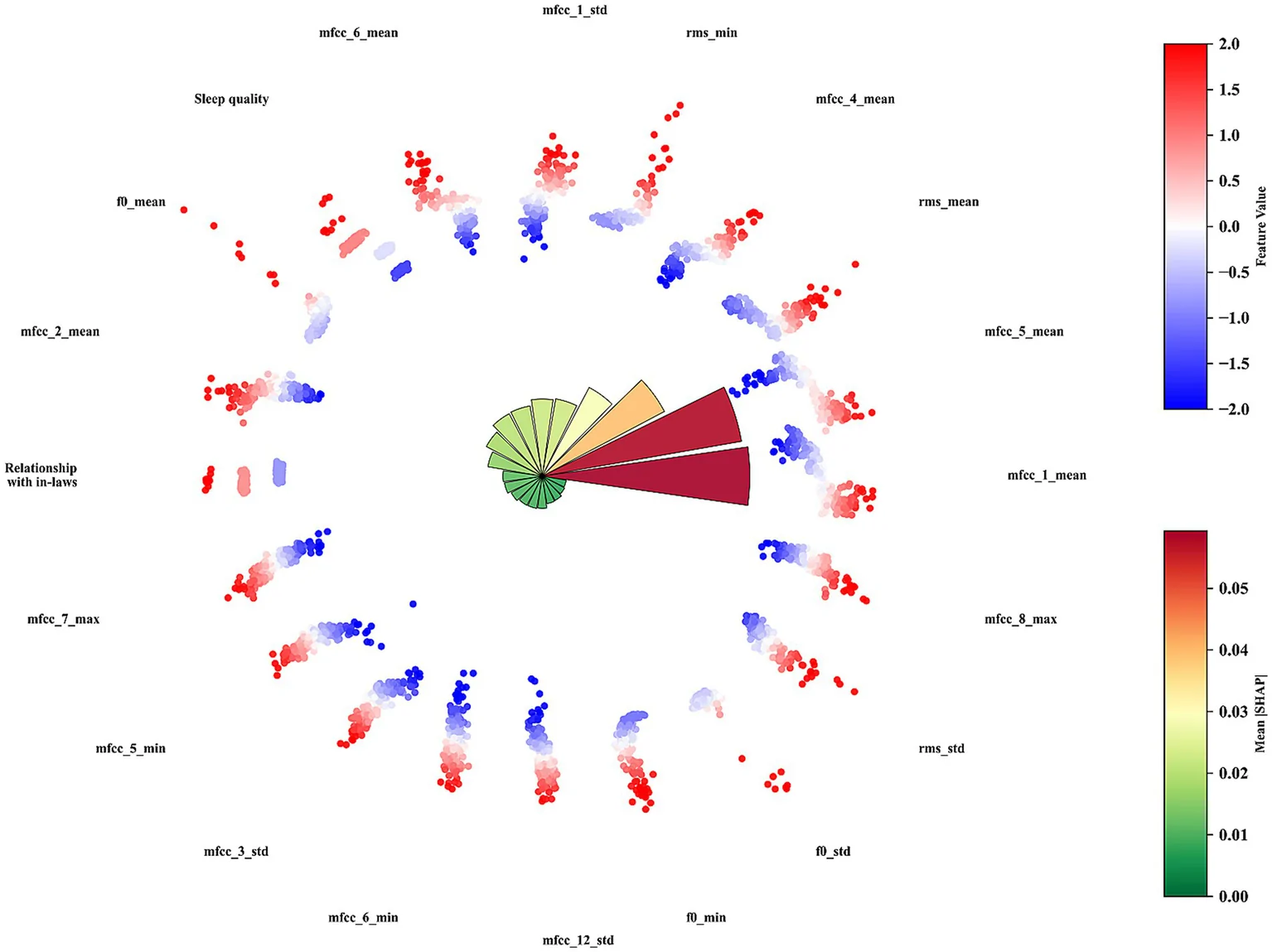
SHAP polar beeswarm plot of the top 20 most influential features in the RF-Fusion model. The inner radial bars represent the global importance of each feature, quantified by the mean absolute SHAP value (darker red indicates higher importance). The outer beeswarm scatter points display the distribution of individual SHAP values across all samples, with the color of each point reflecting the feature value (red: higher feature value; blue: lower feature value). The angular position of each point indicates the SHAP value, where positive SHAP values (counterclockwise deviation) push the model toward predicting PPD screening positivity, and negative SHAP values (clockwise deviation) push toward predicting PPD screening negativity. MFCC, Mel-frequency cepstral coefficient; RMS, root mean square energy; F0, fundamental frequency.

To elucidate the decision-making process of the best-performing balanced model, SHAP (SHapley Additive exPlanations) analysis was conducted on the RF-Fusion classifier. The top 20 most influential features are shown in Figure 3. As a complementary analysis, Gini importance rankings for all seven classifiers are provided in Supplementary Figure S1.

#### 3.3.1 SHAP feature importance of the RF-fusion model

Acoustic features dominated the model’s predictions: 18 of the top 20 features were acoustic, with MFCC-related coefficients accounting for the majority of high-importance positions. MFCC_1_mean exhibited the highest mean absolute SHAP value (0.059), closely followed by MFCC_5_mean (0.058) and RMS_mean (0.039). This indicates that the broad spectral shape of the voice and its loudness characteristics were the most discriminative features. Other prominent acoustic features included MFCC_4_mean, RMS_min, MFCC_1_std, and MFCC_6_mean (Figure 3).

The only two socio-ecological risk factors among the top 20 were postpartum sleep quality (ranked 8th) and relationship with parents-in-law (ranked 11th). Poor sleep quality is a well-documented predictor of PPD (37), and conflict or dissatisfaction with parents-in-law has been consistently identified as a culturally salient risk factor among Chinese postpartum women (38). The identification of these two factors as important predictors in the SHAP analysis is consistent with their established clinical relevance, supporting the validity of the model’s learned representations.

#### 3.3.2 Gini importance across classifiers and feature selection patterns

Several notable patterns emerged from the Gini importance rankings (Supplementary Figure S1). First, the two gradient boosting algorithms (GB and XGBoost) automatically assigned zero importance to five to six features, including several socio-ecological risk factors and HNR mean. This is a known property of gradient boosting, which performs implicit feature selection during training (39). The consequent model sparsity likely contributed to the superior AUCs of these classifiers.

Second, linear classifiers (LR and SVM) distributed importance more broadly across features, with both acoustic and risk factor features receiving non-zero weights. For instance, LR ranked sleep quality, work stress, and multiple MFCCs among its top predictors, while SVM also assigned high importance to sleep quality, work stress, and several MFCC features. This broader feature utilization may explain their relatively lower AUCs compared to tree-based models, as they retained weakly informative or redundant features.

Third, the Naive Bayes classifier exhibited a distinctive pattern, with work stress, ZCR mean, and multiple F0 statistics dominating its top ranks, a configuration not observed in any other classifier. This reflects the inherent property of NB to estimate feature contributions independently, which can amplify the influence of features with strong univariate separation.

Finally, among the acoustic features, MFCC-5 mean, MFCC-4 mean, and RMS mean consistently appeared among the top predictors across all seven classifiers, confirming the robustness of these acoustic markers. The variable rankings observed between SHAP and Gini importance arise from known methodological differences: Gini importance reflects total impurity reduction in tree splits and can overestimate the contribution of continuous or high-cardinality features (40), whereas SHAP values quantify marginal contributions while accounting for feature interactions (41). Given its stronger theoretical foundation, the SHAP-based ranking was used as the primary interpretability metric throughout this study.

### 3.4 Model calibration and clinical utility

**Figure 4:**
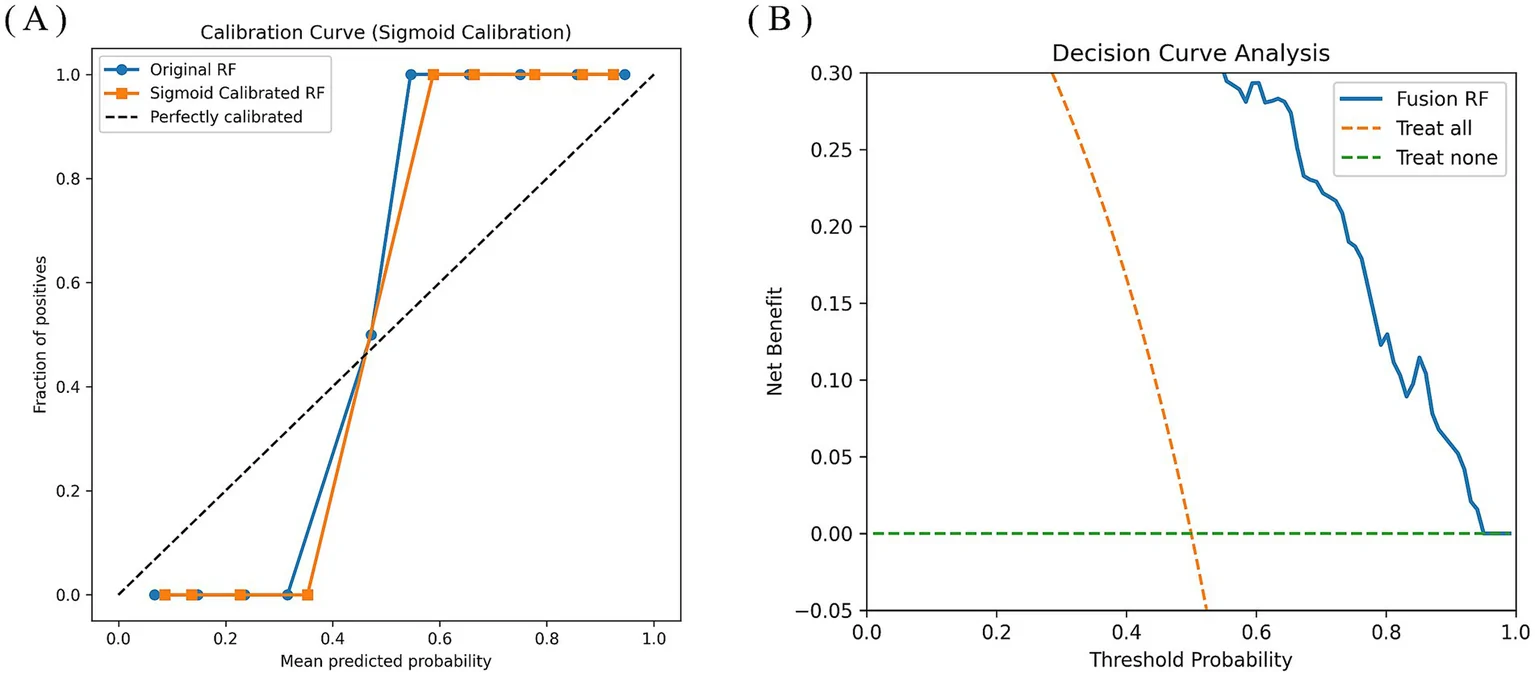
Calibration and decision curve analysis of the RF-Fusion model. **(A)** Calibration curves for the original and Platt-calibrated Random Forest models. The diagonal dashed line represents perfect calibration. Error bars represent 95% confidence intervals. **(B)** Decision curve analysis comparing the net benefit of the RF-Fusion model against the “screen all” and “screen none” default strategies. The x-axis represents the threshold probability for defining a positive screening result. The y-axis represents the net benefit. The model provided a positive net benefit across threshold probabilities ranging from approximately 0.50 to 0.90.

To assess the reliability of the predicted probabilities, calibration analysis was performed on the RF-Fusion model using both the original and Platt-scaled (sigmoid) outputs (Figure 4A). Both models exhibited a similar calibration pattern: calibration was acceptable at low predicted probabilities, showed systematic deviation from perfect calibration in the intermediate range, and aligned with observed risk at high predicted probabilities. The Hosmer–Lemeshow goodness-of-fit test confirmed poor overall calibration for both the original model (*χ^2^* = 32.36, *p* < 0.001) and the Platt-calibrated model (*χ^2^* = 30.37, *p* < 0.001). These results indicate that the predicted probabilities should be interpreted as relative risk indicators rather than precise absolute risk estimates.

Despite the suboptimal calibration performance, decision curve analysis (DCA) demonstrated favorable clinical utility of the model (Figure 4B). The RF-Fusion model achieved a higher net benefit than both the “screen all” and “screen none” strategies across a clinically meaningful range of threshold probabilities (approximately 0.50 to 0.90). This positive net benefit suggests that the model can support clinical decision-making by identifying postpartum women who may warrant further assessment for potential PPD, while reducing unnecessary interventions for those at lower risk.

## 4 Discussion

### 4.1 Principal findings

This prospective short-term longitudinal study developed and systematically compared seven machine learning classifiers to predict PPD screening positivity at 42 days postpartum, using voice acoustic features and socio-ecological risk factors collected on the day before hospital discharge. The principal findings were threefold. First, voice-only models demonstrated consistently better discriminative performance than risk-only models across all classifiers. Second, the fusion of voice and risk factors yielded the highest numerical AUC (0.871 for both RF-Fusion and XGBoost-Fusion), although this improvement over the voice-only models did not reach statistical significance (*p* = 0.147). Third, SHAP analysis of the best-performing balanced model (RF-Fusion) revealed a clear dominance of spectral and energy-related acoustic features in driving model predictions. These results should be interpreted as an initial demonstration of feasibility, given the modest sample size and the absence of external validation.

### 4.2 The predictive value of voice acoustics in the early postpartum period

A central finding was that voice features recorded on the day before hospital discharge could predict PPD screening status at 6 weeks postpartum. The voice-only models achieved AUCs above 0.86, which is noteworthy given the temporal separation between predictor collection and outcome ascertainment. This finding aligns with accumulating evidence that vocal characteristics convey information about a speaker’s emotional and psychomotor state (42). Reduced articulatory precision, diminished vocal energy, and decreased prosodic variability are associated with psychomotor retardation and emotional blunting (32, 43), both of which can present in the early stages of postpartum depressive symptomatology. The performance of voice-only models in this study (AUC up to 0.864) is consistent with findings from prior cross-sectional and longitudinal studies demonstrating the predictive value of acoustic features for depression severity (12, 32). The acoustic features identified as important predictors in this study—MFCCs, F0, and energy-related parameters—are well-established in the broader speech-based depression literature (32, 44). The present contribution, therefore, lies not in the novelty of the acoustic features themselves, but in demonstrating their utility for early screening in a perinatal population using data collected before hospital discharge. While these findings are promising, they should be regarded as preliminary and require confirmation in larger, independent perinatal samples.

### 4.3 The role of socio-ecological risk factors in guiding personalized care

The seven early key risk factors, derived from a socio-ecological framework, demonstrated meaningful discriminative power when used alone (GB risk-only AUC = 0.825), corroborating their established clinical importance in PPD. In the fusion models, the incremental gain in AUC was modest, and only postpartum sleep quality and relationship with parents-in-law appeared among the top 20 SHAP features. This pattern, however, should not be interpreted as evidence that the remaining risk factors lack clinical value.

Rather, the acoustic signal and the risk factors serve distinct and complementary functions in perinatal care. Voice features provide an efficient, objective means of identifying women who may be at elevated risk. The socio-ecological risk factors, by contrast, provide granular information on what specific domains may be driving that risk for a given individual. For instance, a woman identified by the voice model as high-risk may have her risk attributable primarily to poor sleep quality, whereas another may be experiencing significant interpersonal distress with her parents-in-law. These two women require different, tailored nursing interventions: the former may benefit from sleep hygiene counseling, while the latter may require family-based psychoeducation or referral to social work services. In this sense, the voice-based model serves as the first stage of a two-stage screening process, while the risk factor profile guides the content of subsequent care.

This pattern should not be interpreted as diminishing the clinical value of these risk factors. Rather, it suggests that the acoustic signal may already capture the downstream physiological and psychomotor consequences of these factors. Sleep disruption can affect vocal fold function, respiratory control, and muscle tone (45), while interpersonal distress with parents-in-law—a culturally salient stressor among Chinese postpartum women—may increase vocal tension and reduce prosodic variability (38, 46). From this perspective, voice acoustics serve as an objective, integrative readout of the cumulative impact of diverse psychosocial stressors on the maternal biobehavioral system during the early postpartum period.

### 4.4 Comparison with prior work

The voice-only AUC of 0.864 (XGBoost) and the RF-Fusion AUC of 0.871 observed in this study fall within the performance range reported in the automatic speech analysis (ASA) literature. A recent systematic review by Briganti and Lechien (47) found that speech and voice quality measures consistently distinguished depression from controls, with AUCs ranging from 0.71 to 0.93 and classification accuracies from 78 to 96.5%. The present findings, obtained with standard classifiers and handcrafted acoustic features, are consistent with this evidence base. In terms of other classification metrics, a recent meta-analysis of 105 ASA studies reported pooled mean highest accuracy of 0.81, sensitivity of 0.84, and specificity of 0.83 (11). The RF-Fusion model in the present study achieved an accuracy of 0.825, a sensitivity of 0.804, and a specificity of 0.845. Formal statistical comparison between the fusion and voice-only configurations did not indicate a significant improvement (*p* = 0.147), suggesting that the acoustic features alone account for the majority of the model’s discriminative power.

The present study extends prior work by focusing on a Chinese postpartum population, a group that has received limited attention in voice-based mental health screening research, and by demonstrating that data collected before hospital discharge can predict PPD screening status 6 weeks later, addressing the underexplored potential of voice-based screening in perinatal mental health.

### 4.5 Clinical utility for early identification and personalized intervention

Calibration analysis indicated that both the original and Platt-scaled RF-Fusion models showed systematic deviation from perfect calibration (Hosmer-Lemeshow test, *p* < 0.001). The predicted probabilities should therefore be interpreted as relative risk indicators rather than precise absolute risk estimates, and the model is best understood as a screening triage tool rather than a diagnostic instrument. Despite this limitation, decision curve analysis demonstrated that the model provided a positive net benefit across threshold probabilities of approximately 0.50 to 0.90, outperforming both the “screen all” and “screen none” strategies. This suggests that the model, even without perfect calibration, can meaningfully stratify postpartum women by risk before discharge. However, given the preliminary nature of these findings, the model should be regarded as a proof-of-concept rather than a tool ready for clinical deployment.

Beyond risk stratification, the seven early key risk factors included in the fusion model offer clinically actionable information to guide individualized postpartum care. Four of these factors—sleep quality, relationship with parents-in-law, social support, and appraisal of the postpartum recuperation environment—were rated on a five-point Likert scale, while the remaining three—work-related stress during maternity leave, adequacy of spousal caregiving, and distress over gestational weight gain—were assessed as binary items (yes/no). Sleep quality and relationship with parents-in-law emerged among the top 20 SHAP features, suggesting that these domains may be particularly reflected in vocal characteristics. However, the other risk factors, though contributing less to the model’s overall predictive performance, retain clinical value as they capture distinct and potentially modifiable stressors known to be associated with postpartum depressive symptoms (48, 49). For instance, a woman reporting inadequate spousal caregiving may benefit from couple-based psychoeducation, while a woman distressed by gestational weight gain may require nutritional counseling and body image support. By combining automated voice-based screening with a structured risk factor profile, clinical nurses can move beyond a single screening score toward a two-tier care pathway: risk detection followed by individualized intervention planning.

Based on the present findings, a practical implementation pathway can be envisioned. In routine postpartum nursing workflows, a brief picture description task could be administered via a tablet device during the discharge education session. The voice sample would be processed in real time by a locally deployed, lightweight model—such as Random Forest with the top 10 acoustic features validated in our sensitivity analysis—to generate an immediate risk score. Women identified as high-risk could be referred to a structured diagnostic interview or scheduled for an earlier postpartum mental health follow-up, while women at low risk would continue with routine care. This approach would shift the timing of PPD screening from the 42-day visit to before hospital discharge, enabling earlier risk identification. However, the present model should be regarded as a proof-of-concept. Multi-site validation studies, prospective implementation trials, and assessments of acceptability to both postpartum women and healthcare providers are necessary prerequisites for clinical deployment.

### 4.6 Limitations

Several limitations should be acknowledged. First, the sample size (64 participants, 192 speech samples) is modest relative to the complexity of the analytical approach. In machine learning, small samples can lead to increased variance in performance estimates and may reduce the stability of feature importance rankings across cross-validation folds. Although the use of GroupKFold cross-validation mitigates this concern by ensuring that all samples from a given participant are strictly confined to the same fold, the generalizability of the reported model performance metrics to broader perinatal populations remains to be established. Therefore, the present findings should be interpreted as an initial demonstration of feasibility, and external validation in larger, multi-site perinatal cohorts is essential before clinical translation can be considered. Second, PPD status was determined using the EPDS, a validated screening instrument, rather than a structured clinical interview. While the EPDS is the most widely used PPD screening tool globally, it does not constitute a formal clinical diagnosis, and the potential for outcome misclassification should be noted. Third, the suboptimal probability calibration (Hosmer–Lemeshow test, *p* < 0.001) suggests that predicted probabilities should be interpreted as relative risk indicators rather than precise absolute estimates. Fourth, several methodological choices limit generalizability. The balanced dataset construction may not fully reflect screening performance in a naturally imbalanced population. The single-site design in Qingdao, China, limits cultural and demographic generalizability, and subtle variability in recording conditions across participants may have introduced unmeasured variance into the acoustic measurements. The HNR was computed only as a global mean, and the picture-description task may not capture the full range of spontaneous affective expression. Finally, although the sample-to-feature ratio may raise concerns about overfitting, a sensitivity analysis using only the top 10 acoustic features (Supplementary Table S1) demonstrated comparable performance, indicating that the core discriminative information is concentrated in a small number of robust features. Multi-site studies across diverse perinatal settings with larger, unselected cohorts are needed to evaluate the robustness and generalizability of voice-based PPD screening.

### 4.7 Future directions

Future research should validate the current findings in larger, more diverse perinatal populations and investigate whether the model’s early prediction capability extends to other postpartum outcomes, such as anxiety and maternal–infant bonding difficulties. The incorporation of dynamic acoustic features and deep learning-based representations may further enhance predictive performance. Implementation studies are also warranted to evaluate how a voice-based early screening tool can be integrated into routine discharge workflows and to assess its acceptability to both postpartum women and healthcare providers. An important consideration for future clinical translation is the robustness and cross-domain generalizability of voice-based screening models. Recent work has highlighted that model performance can be substantially influenced by recording conditions, elicitation protocols, and population characteristics (44). Cross-linguistic generalization represents an additional challenge, as most speech-based depression models have been developed and validated in Western, English-speaking populations (11, 42). Furthermore, the successful application of voice-based risk stratification in real-world telehealth settings (50) suggests that ecological validity can be maintained outside controlled laboratory environments, provided that data collection and model development follow rigorous, standardized protocols (11, 44). Multi-site studies employing diverse recording environments, varied elicitation tasks, and demographically heterogeneous perinatal samples are therefore needed to evaluate the generalizability and clinical scalability of voice-based PPD screening before widespread implementation.

## 5 Conclusion

This prospective short-term longitudinal study developed and compared seven machine learning classifiers for early prediction of PPD screening positivity, using voice acoustic features and socio-ecological risk factors collected on the day before hospital discharge. Voice features alone demonstrated strong predictive performance; the addition of risk factors yielded a marginal, non-significant improvement (*p* = 0.147). Spectral and energy-related acoustic features were the dominant contributors to model predictions. Despite imperfect calibration, decision curve analysis supported the clinical utility of the model for risk stratification. These findings provide preliminary evidence that voice acoustic features may serve as objective markers for early PPD screening. However, external validation in larger, independent cohorts and prospective implementation studies are necessary before clinical application can be considered.

## Data Availability

The raw data supporting the conclusions of this article will be made available by the authors, without undue reservation.
