# Supplementary material for: A multimodal feature fusion model integrating voice acoustic features and early key risk factors for early screening of postpartum depression: a prospective short-term longitudinal observational study
Source: Front Public Health. 2026 Aug 25;14:1906621. doi: 10.3389/fpubh.2026.1906621 (PMC13549822; doi:10.3389/fpubh.2026.1906621)
Supplement: Supplementary file 1 [file Data_Sheet_1.PDF]

Supplementary Figure S1. Top 10 feature importance rankings for the seven base classifiers based on Gini importance (or equivalent metrics).

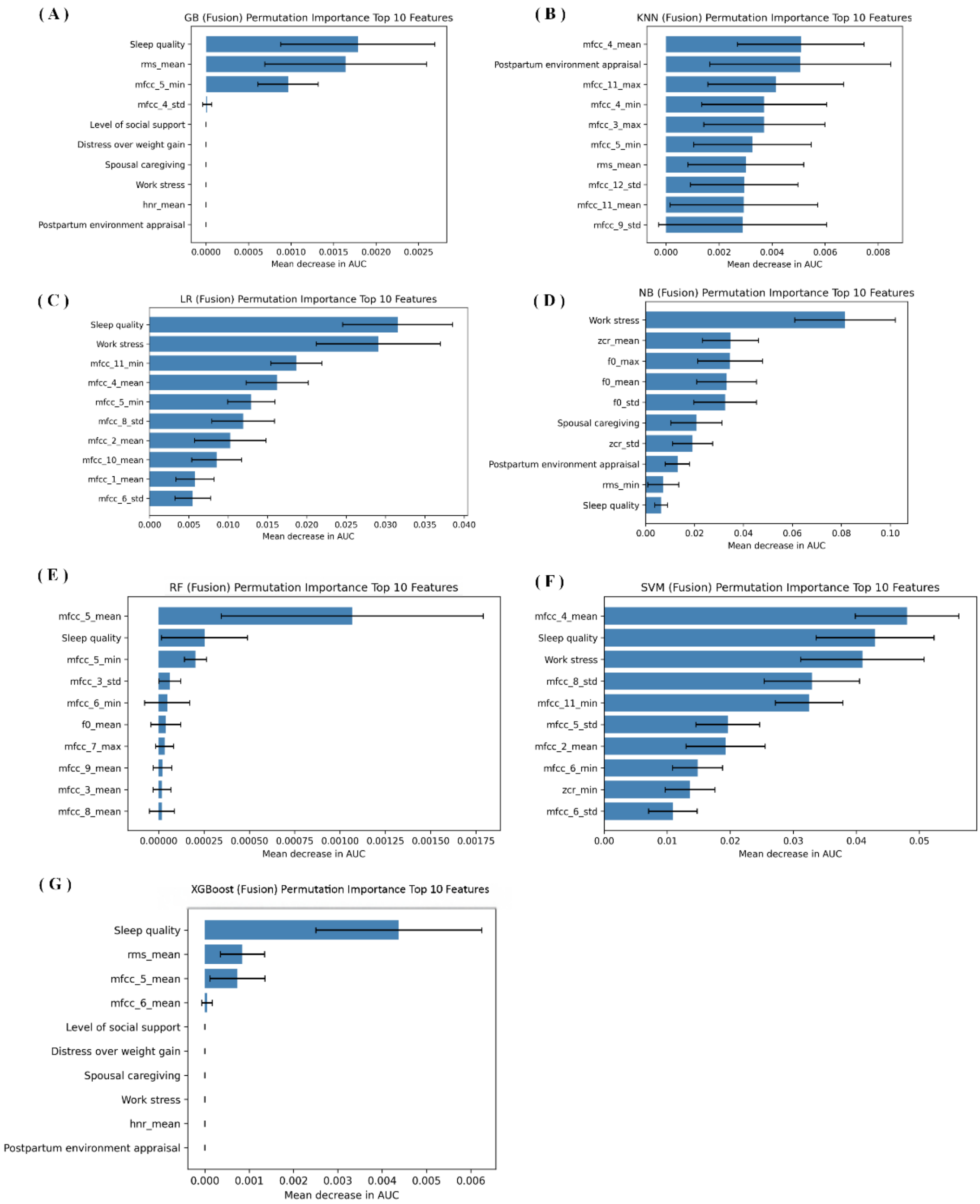

The figure presents the top 10 most influential features for each of the seven classifiers: (A)Gradient Boosting (GB), (B)K-Nearest Neighbors (KNN), (C)Logistic Regression (LR), (D) Naïve Bayes (NB), (E) Random Forest (RF), (F) Support Vector Machine (SVM), and (G) eXtreme Gradient Boosting (XGBoost). Feature importance is measured by the Gini impurity decrease for tree-based models (GB, RF, XGBoost). For non-tree models (KNN, LR, NB, SVM), equivalent importance metrics (e.g., absolute coefficients for LR, permutation-based

importance for others) are used to ensure comparability under the same “Gini-like” ranking convention. Longer bars indicate higher importance. This complementary analysis helps to compare the feature preferences across different algorithmic families and supports the main findings based on the best-performing balanced model (RF-Fusion) shown in Figure 3.
