# Supplementary material for: A multimodal feature fusion model integrating voice acoustic features and early key risk factors for early screening of postpartum depression: a prospective short-term longitudinal observational study
Source: Front Public Health. 2026 Aug 25;14:1906621. doi: 10.3389/fpubh.2026.1906621 (PMC13549822; doi:10.3389/fpubh.2026.1906621)
Supplement: Supplementary file 2 [file Table_1.DOCX]

**Supplementary Table S1. Sensitivity analysis using the top 10 acoustic features only.**

| **Model** | **AUC (95% CI)** | **Accuracy (95% CI)** | **Sensitivity (95% CI)** | **Specificity (95% CI)** | **F1 (95% CI)** |
| --- | --- | --- | --- | --- | --- |
| LR | 0.841 (0.783–0.893) | 0.757 (0.698–0.818) | 0.774 (0.687–0.852) | 0.739 (0.653–0.825) | 0.759 (0.691–0.820) |
| SVM | 0.843 (0.782–0.895) | 0.762 (0.703–0.818) | 0.784 (0.700–0.860) | 0.741 (0.659–0.828) | 0.766 (0.699–0.828) |
| **RF** | **0.891 (0.840–0.932)** | **0.825 (0.771–0.875)** | **0.794 (0.713–0.868)** | **0.857 (0.784–0.923)** | **0.819 (0.754–0.873)** |
| KNN | 0.885 (0.836–0.928) | 0.809 (0.755–0.865) | 0.783 (0.701–0.863) | 0.836 (0.756–0.909) | 0.803 (0.740–0.861) |
| NB | 0.884 (0.827–0.929) | 0.824 (0.766–0.875) | 0.741 (0.645–0.825) | 0.907 (0.850–0.962) | 0.807 (0.736–0.865) |
| GB | 0.864 (0.806–0.916) | 0.804 (0.740–0.859) | 0.813 (0.726–0.890) | 0.794 (0.713–0.874) | 0.804 (0.736–0.865) |
| XGBoost | 0.871 (0.817–0.919) | 0.793 (0.734–0.844) | 0.793 (0.711–0.865) | 0.793 (0.713–0.874) | 0.792 (0.726–0.848) |

Notes: The top 10 acoustic features were identified by the SHAP analysis of the RF-Fusion model (Figure 3) and included: MFCC_1_mean, MFCC_5_mean, RMS_mean, MFCC_4_mean, RMS_min, MFCC_1_std, MFCC_6_mean, F0_mean, MFCC_2_mean, and MFCC_7_max. All metrics were evaluated under 10-fold GroupKFold cross-validation. 95% confidence intervals (in parentheses) were estimated via bootstrap (1000 resamples). Abbreviations: LR, Logistic Regression; SVM, Support Vector Machine; RF, Random Forest; KNN, K-Nearest Neighbors; NB, Gaussian Naive Bayes; GB, Gradient Boosting; AUC, Area Under the Receiver Operating Characteristic Curve; CI, Confidence Interval.
